# Three-dimensional view of ultrafast dynamics in photoexcited bacteriorhodopsin in the multiphoton regime and biological relevance

**DOI:** 10.1038/s41467-020-14971-0

**Published:** 2020-03-06

**Authors:** R. J. Dwayne Miller, Olivier Paré-Labrosse, Antoine Sarracini, Jessica E. Besaw

**Affiliations:** 1 Max Planck Institute for the Structure and Dynamics of Matter, Luruper Chaussee 149, 22761 Hamburg, Germany; 2 Departments of Chemistry and Physics, University of Toronto, 80 St. George Street, Toronto, ON M5S 3H6 Canada

**Keywords:** Biochemistry, Biophysics, Structural biology, Biochemistry, Photochemistry

## Abstract

How does chemistry scale in complexity to unerringly direct biological functions? Nass Kovacs et al. have shown that bacteriorhodopsin undergoes structural changes tantalizingly similar to the expected pathway even under excessive excitation. Is the protein structure so highly evolved that it directs all deposited energy into the designed function?

It is difficult to overstate the importance of having atomic structures to help shape our thinking and understanding of matter. Structural information constrains the number of possible solutions in trying to piece together a puzzle in how matter undergoes transformation from one structure to another and the associated changes in material properties^1,2^. In terms of understanding biological processes, this question always reduces to how the protein structure surrounding an active site has evolved to direct chemical processes into biological functions, typically with efficiencies well beyond our current capabilities to exploit chemistry.

In this respect, bacteriorhodopsin (bR) serves as a model system for understanding structure-function relationships for membrane proteins^3–5^. This system functions as a light-driven, outward proton pump, which can be triggered by light to use time resolved optical methods to watch it function in real time. Its structure is composed of seven transmembrane α-helices that are covalently bound to a photoactive retinal molecule via a lysine residue through a Schiff base linkage (Fig. 1b). Upon absorbing a photon, the retinal chromophore undergoes rapid isomerization from an all-*trans* to 13-*cis* form passing through the I_460_ (charge separated), J_625_ (highly twisted) and K_590_ (isomerized) intermediates. The retinal isomerization acts like a push in changing the electrostatic and structural environment around the active site. These changes in turn lead to a series of cascaded protein conformational changes to facilitate the transport of a proton from the retinal Schiff base to the extracellular side of the membrane via L_550_ and M_410_ intermediates. The retinal then undergoes reprotonation and thermal re-isomerization through the N_560_ and O_630_ intermediates, respectively, where it can then return to the bR_568_ ground state. These processes have been well characterized spectroscopically and many of the long-lived structural intermediates determined. However, we can only guess about the structural details leading to the initial push that sets these changes into motion. There is an enormous lesson to be learned from this system—if only we could watch it work at the atomic level to capture the primary events driving the proton pump.

**Fig. 1:**
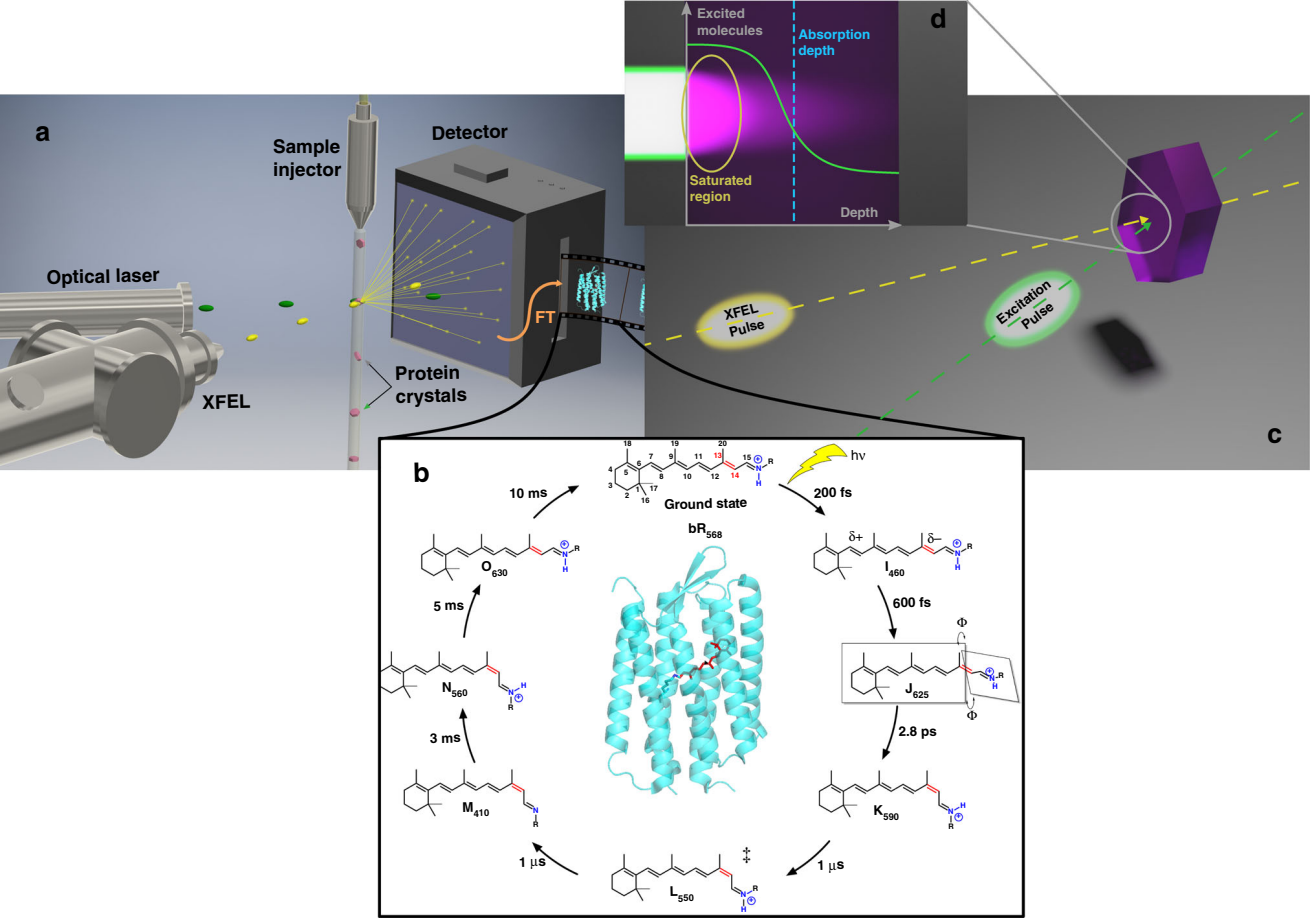
Making molecular movies and excitation considerations. **a** Time-resolved serial crystallography is schematically shown using a lipid cubic phase injector per ref. ^3^. Femtosecond optical laser pulses (532 nm) initiate the photocycle in a train of bR crystals with random orientations. The fs X-ray (XFEL) pulses capture the photoinduced structural changes by diffraction that are Fourier Transformed (FT) to real space structures to make a movie of the structural changes. **b** The photocycle of retinal in bacteriorhodopsin (bR) captured in the “molecular movie” film in the single photon regime (refer to text for details). Ground state bR (PDB code: 1C3W) is depicted in the center with retinal covalently bound to bR though a Schiff base linkage to a conserved lysine residue. The source of the transported proton is the protonated retinal Schiff base, highlighted in blue. The key isomerization step from all-*trans* to 13-*cis* retinal occurs about the C13=C14 bond, accented in red. ^‡^Subsequent, protein structural changes are occurring in the ps–ms time region in order to facilitate proton transport. **c** The time base for the movies is determined by the speed of light and variable pathlength difference as shown between the fs excitation and fs X-ray probe pulses to give <100 fs time resolution to atomic motions. **d** The inset shows the mismatch between the laser excitation, which is strongly absorbed, and the X-ray pulse, which samples the entire crystal thickness (10 µm scale). Excitation well above 1-photon per photoactive chromophore is often used to try to excite a larger fraction of the probed volume to decrease unexcited background scatter from obscuring the photoinduced structural changes. At such high fluences, saturation effects occur (shown) that also lead to fully resonant coherent 2-photon, sequential resonant 2-photon, nonresonant 2-photon, to n-photon transitions depending on the laser pulse width and associated peak power. Crystals on the order of the 1/*e* absorption depth (dashed blue line) should be used for maximum contrast above background under conditions to ensure 1-photon absorption to well defined excited states (see text).

It is now possible to do exactly this with either electron or X-ray sources^1,2^. The experimental protocol involves taking a time series of movie frames depicting the structural change, captured as diffraction images, much like a movie camera based on film where in this case the protein crystals are the movie film (Fig. 1). The structural changes need to be triggered by femtosecond (fs) lasers to provide a short excitation (“start”) pulse that is in turn synchronized with electron or X-ray probe (“stop”) pulses with equally short durations (order 100 fs or 10^−13^ s) that act as an ultrafast shutter at various time delays to capture the atomic motions in a stroboscopic fashion. The information one can obtain on the reaction dynamics of interest is only as good as the knowledge of how you triggered the reaction. The laser excitation step defines this condition (see Fig. 2). Excitation to different excited states than the biologically relevant excited state leads to different reaction pathways and reaction forces that would affect at, some level, what motions are observed and magnitudes.

**Fig. 2:**
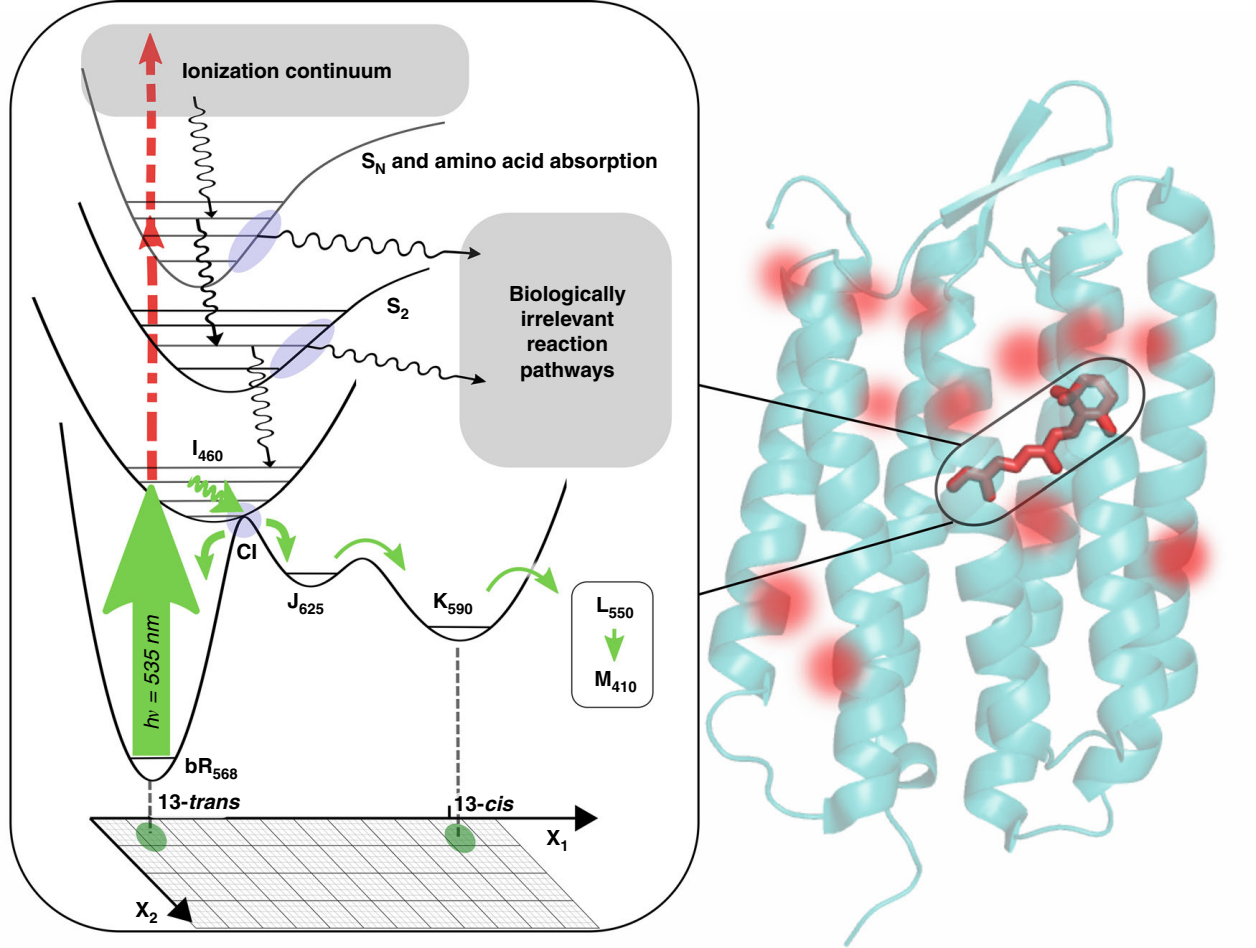
Photophysical and chemical pathways at high peak power and fluences. The seven transmembrane helical structure of bR is shown with retinal identified by the solid red structure. The red hot spots denote the positions for nonresonant two-photon excitation of Trp at high peak powers, which would be random and not contribute coherently to changes in diffraction (per ref. ^3^). However, all the resonant multiphoton pathways involving retinal would occur at the same location in space, i.e. at the retinal site, and contribute to photoinduced changes in diffraction. The left panel shows the biologically relevant one-photon pathway (solid green) for photoisomerization of retinal in relation to the multiphoton pathways (dashed green). The functionally relevant motions are highly constrained by the protein structure in going from the initially formed photoisomer, which is strongly coupled to distinct retinal-protein conformational states (I-M). In contrast, the different multiphoton pathways to different excited state surfaces have relaxation channels (black wiggly arrows) to photoproducts in undefined reaction coordinate space (x_1_, x_2_) unrelated to biological function. The need to excite the biologically relevant one-photon pathway is clear. This figure shows the importance of exciting in the one-photon, linear response, regime (see text for guidelines).

The recent report by Nass Kovacs et al.^3^ used this approach exploiting the high brightness and femtosecond time resolution capabilities of X-ray Free Electron Lasers (XFELs) to study bR with sufficient space-time resolution to resolve a wealth of structural changes. One of the biggest open questions is how a molecule with over a 100 vibrational degrees of freedom can channel the absorbed photon energy to selectively isomerize at the C13-C14 position of the retinal (Fig. 1b). This process is kick-started by an initial displacement of charge density in the excited state that leads to bond softening and elongation with increased torsional motion about this bond and accompanying conformation changes in protein around the active site. These long guessed details are now observed as well as important newly resolved motions of interstitial waters. These findings are similar to a previous report with noted differences^3,5^.

## The peak power problem

The most important aspect of this work is the rigor to which they characterized the excitation typically used in XFEL experiments. The authors studied not only the power dependence using multiple spectral markers to quantify nonlinear changes in photoproduct formation, but also determined the scattering (<20%) from their lipid cubic phase crystal delivery system. With the corrected incident fluence, they established that they used 36 photons per retinal within the 1/*e* absorption length of the bR crystals. This detail alone clearly establishes that the experiments were conducted in the multiphoton regime. The corresponding peak power for their excitation pulse durations was 500 GW/cm^2^ where the onset for nonlinear behavior occurs around 30 GW/cm^2^, in which case the initial state preparation is ill defined (see Fig. 2 of ref. ^3^). This finding has implications for all previous studies conducted using similar peak powers or fluences^5–9^.

The authors made the case, based on theoretical calculations, that the clearly observed multiphoton processes in their peak power control studies were attributable to non-resonant two-photon absorption by the tryptophan (Trp) residues within the retinal binding site, i.e., the transition requires the simultaneous action of two photons at the excitation wavelength of 535 nm to have sufficient energy to excite Trp. This alone illustrates how high the peak power was. It was argued these excitation processes are inconsequential with respect to excitation of retinal. However, we estimate that the fully resonant 2-photon absorption of retinal at 535 nm is at least 2 orders of magnitude larger than the nonresonant process for Trp^10,11^. This estimate is further reinforced by the observed saturation of the non-resonant 2-photon absorption (800 nm) of retinal (bR) at 700 GW/cm^2^—to give a completely different photoproduct—that is specifically exploited for optical memory^12,13^. The fully resonant 2-photon absorption of retinal (at 535 nm) would saturate at more than an order of magnitude lower peak power than observed at 800 nm, approximately where the authors see the onset of nonlinear behavior (>30 GW/cm^2^, Fig. 2 of ref. ^3^), and lead to even higher multiphoton processes at higher powers. These resonant two-photon and higher processes of retinal access higher-lying electronic states, which can explore many different potential energy surfaces that lead to different photoproducts than the biologically relevant S_1_ excited state channel (see Fig. 2).

## Biological relevance: pathway confusion

The most important fundamental point to come out of this work is that despite all the possible different photochemical pathways sampled from high lying electronic states accessed via multiphoton absorption, there is a remarkable convergence at long time (ms) to the previously assigned M structure^14^. It seems that no matter the starting point, the system is poised to channel energy into forming the structural intermediates most intimately involved in the key proton transfer step.

This inadvertent discovery could end up being extremely important in trying to understand structure-function relationships of biological systems. It may well be that there is a multi-tiered optimization process at work. The surrounding protein matrix has clearly been optimized to selectively direct trans to cis isomerization at the C13-C14 position in the S1 excited state, which subsequently triggers a cascade of thermally sampled processes leading to proton translocation—the biological purpose for energy storage. There are still multiple, irrelevant, conformational pathways for both the retinal and surrounding protein possible at each step along the relaxation cascade that could negatively affect the water network and proton transport within the perturbed protein structure. There are many more substates and much longer time to sample such meandering pathways for the subsequent I to M intermediate steps than the ultrafast primary processes. There would need to be constraints on the fluctuations to keep the process focused as additional tiers of optimization. We may well be seeing our first evidence of just how strongly correlated the motions of the retinal and protein surroundings are to keep the reaction focused from femtoseconds to milliseconds. We would not have this insight without excessively “kicking” the retinal (via multiphoton processes) and still getting the same result as the biologically relevant excitation.

There is, however, a caveat. The referenced “M” structure^14^ was also determined using similarly high fluences, with >20 photons per retinal in the 1/*e* absorption depth (Fig. 1d), such that the total excess energy deposited into the protein by sequential multiphoton and associated temperature jump (order 100 °C, µs-ms decay)^15,16^ will be comparable. In this case, this coincidence could be expected as a consequence of thermally sampled pathways—not photochemically driven.

## Concluding remarks

We will not be able to discern the level of organization and degree of dynamic coupling between the retinal and the protein structure until we determine the structural changes under biologically relevant conditions. To ensure one-photon processes to relevant excited states, the excitation fluence must be less than one photon per photoactive chromophore within the 1/*e* absorption depth (ideally less than 0.2 photons to give <10% contribution from 2-photon channels), and normalized peak power dependences of transient spectra need to be done to confirm linear response (typically <100 GW/cm^2^).

It is the magnitude of the motions and correlations through space that define how the chemistry, in this case isomerization or a simple push, is transduced into biology. The pathway matters. This work is a significant step forward in putting us on the right track.
